# A Comfort for Invalids

**Published:** 1870-08

**Authors:** 


### A COMFORT FOR INVALIDS.

Sometimes the sick are much annoyed at night by the light in the room attracting insects, giving an unpleasant odor, or vitiating the air of the chamber. Take any kind of box, remove the cover, set it up lengthwise, place a lamp or candle in it, and fasten it on the outside of the window so as to set close to it, with the open part looking through the window into the room.

